# Managing Major Life Changes: An Exploratory Study Using the Bridges Transitions Framework to Help Foster Youth Prepare for Discharge

**DOI:** 10.3390/children12010022

**Published:** 2024-12-26

**Authors:** Ande A. Nesmith

**Affiliations:** School of Social Work, University of St. Thomas, St. Paul, MN 55105, USA; nesm3326@stthomas.edu

**Keywords:** foster youth, transitions to adulthood, Bridges Transitions Framework, youth transitions

## Abstract

Background: Adolescents in foster care endure frequent disruptive transitions, often culminating in discharge to independent living rather than reunification or adoption. Former foster youth fare poorly once on their own, with high rates of homelessness and social disconnection. This study explored the use of the Bridges Transitions Framework near the end of placement to help youth cope with the transition to adulthood. Methods: In this exploratory study, the framework was integrated into a foster agency’s programming; then, we assessed outcomes using administrative data and youth interviews. Thirty-five youth participated. Status of employment, education, and social support was collected 18 months after exposure to the framework. Results: The participants reported moderate to high levels of social support, which is often limited or absent among foster care leavers. Relative to rates reported in state-level foster care data, participants had substantially higher rates of school enrollment after discharge. With very few empirically assessed models available for this population that specifically address internal coping with such substantial life changes, the Transitions Framework offers a tool that may help foster youth navigate aging out of care. Securing lasting and meaningful social support and employment and completing education remain elusive for former foster youth. Conclusions: To confirm the utility of the Transitions Framework, it is recommended to assess it with a large sample and matched comparison group over time.

## 1. Introduction

Older youth in child welfare placements experience high rates of living situation changes throughout their foster care stays, culminating in discharge from care [1,2]. The first transition from their family home into foster care can be traumatic in the life of adolescents, who are still developing their sense of identity [3].

One of the first in-depth and well-designed prospective longitudinal studies of older foster youth and their transitions to adulthood was conducted in the U.S. Midwest: the “Midwest Evaluation of the Adult Functioning of Former Foster Youth”, known as “The Midwest Study” [4,5]. Although their work began nearly twenty years ago, the findings continued to be used and referenced widely and have since been replicated in other regions [6]. Courtney and colleagues’ research found that placement moves during foster care can have a cumulative effect on youth once they transition out of foster care into independent living, impacting education, employment, housing stability, and social support in early adulthood [4,5]. Similar findings have been found in other studies [7,8]. This represents no small subset of foster children. In the U.S., 23% of children enter care as adolescents (13+ years), and 28% of all children currently in care are placed during their adolescent years [9]. Rather than being reunified with their families or adopted, as many as 9% of youth who leave the system each year do so by aging out of the system [9]. The impact of placement into care is long-lasting and may be compounded by additional changes that occur during placement spells (especially moves to different homes) [10,11]. Social service providers may be faced with helping youth cope with their admission into care and transitions during placement, while simultaneously preparing them for the transition out of care.

U.S. federal policies require that the child welfare system attends to the impact of these major disruptions at both admission and discharge. For example, the Fostering Connections to Success Act (2008) [12] prioritizes stable kin relationships and connections to original communities and schools when children are first placed. It requires that, when possible, children remain in their current schools to enhance educational stability. Looking toward discharge, the Foster Care Independence Act (1999) [13] appropriates substantial funds to independent living programs to provide education, training, employment services, and financial support for foster youth aged 16 to 18. However, as will be discussed here, adhering to these policies has proven challenging in practice, and outcomes for former foster youth continue to be poor.

There is very little research evidence that points to improved outcomes for youth aging out of foster care [14,15]. The California Evidence-based Clearinghouse for Child Welfare [14] examines and rates programs for scientific evidence of positive outcomes. In their category regarding youth transitioning to adulthood to live independently, only one program was identified as being supported by scientific evidence. Three programs were described as having promising evidence, and the remainder could not be assessed. The program with scientific evidence, Better Futures, sought to increase post-secondary education among those who were not opposed to continuing their education by providing a 3-day training, community-based coaching, and relationship support. Greeson and colleagues (2020) [15] examined 79 programs and interventions for youth aging out and found only three that were supported with robust research evidence that they affected outcomes within six months to a year post-intervention. These focused on mental health, post-secondary education, and/or building supportive relationships.

Absent from most of these interventions is attention to the impact of major life changes on the youth, their internal response to them, and external reactions that, without some guidance, may lead to behaviors that can make such transitions difficult. The present study explores the application of a framework designed specifically to help individuals cope with difficult life transitions. This study explores the utility of the Bridges Transitions Framework [16] to help older foster youth navigate transitions and determine if there were signs of improved education, social support, or employment at least in the short-term—18 months later.

### 1.1. Transitions While in Foster Care Placement

Those who enter foster care as adolescents typically undergo several placement transitions until they exit the system, most often by reaching the age of majority rather than by adoption or family reunification [2]. The earliest moves tend to be among foster homes; however, with continued disruptions, youth placements increasingly transition away from family-like settings and into congregate care, group homes, residential treatment centers, or juvenile justice settings. In fact, among those entering care at age 13 or older, about half will move from family-like settings into congregate care [17]. This can be a self-perpetuating situation because placement instability can also exacerbate existing behavioral issues [18,19].

Youth in foster care often have limited control over important decisions such as placement moves, which can affect their ability to cope with substantial transitions [10,20]. These decisions represent major life changes such as admission to foster care or moves to new placements, neighborhoods, and schools, yet youth often have little say in them. In turn, high rates of moves can negatively impact education, employment, housing, and social support.

### 1.2. Education

The more moves a youth experiences, the more likely their education is also disrupted, which can have a lasting impact that extends into adulthood [2,21]. Despite federal law, the necessary resources and coordination between schools and child welfare organizations are frequently lacking. Transcripts and Individual Educational Plans (IEPs) often do not transfer with placement moves, slowing academic progress and contributing to the low high school graduation rates observed among foster youth [21]. Moreover, the level of educational attainment decreases with each additional move during a foster care spell. By age 23, about a quarter of former foster youth still do not have a high school diploma or GED [5].

### 1.3. Employment and Housing

Former foster youth struggle to secure employment and housing. Independent living services that coach foster youth in concrete skills such as money management or resume and job interviewing training are essential but not enough to bring stability to these youth post-discharge [22]. Courtney and colleagues’ (2005) [4] Midwest Study found that fewer than half of discharged foster youth were employed by age 19. This is also true on a broader level: the National Youth in Transition Database reveals that, among former foster youth across the U.S., only 35% are employed at least part-time [9].

Stable housing remains elusive for many former foster youth. In the years following discharge, periods of homelessness are common. By 19 years old, 19% of former foster youth experienced at least one episode of homelessness nationally, and 32% experienced at least one episode of homelessness in the same state as the present study [9,20].

### 1.4. Social Support

Independent living services also do not adequately address the socio-emotional needs of youth [16,23]. A growing body of research points to the critical role of social and emotional support—particularly from a supportive adult—that any young person needs during transition to adulthood, yet this is minimal or absent for foster youth [11].Transitions during placement can impact the support youth are able to secure as they exit care [24]. Recurring placement moves can result in a sense of rejection, especially when they occur repeatedly. Youth may respond by “emotionally shutting down”, withdrawing, or behaving in ways that increase the chances of another transition. This, in turn, can further impede their ability to connect with future caregivers or supportive adults [10]. These are among the behaviors that the Transitions Framework aims to ameliorate.

This study examined employment, social support, and education among young people who were placed in foster care as adolescents 18 months after they were trained in applying the Transitions Framework to their own lives.

### 1.5. Conceptual Framework: Bridges’ Transitions

The Transitions Framework [16] draws a distinction between external changes one experiences and internal transitions that are responses to those changes. Change is situational, typically events that occur outside an individual’s control, such as the death of a loved one, losing a job, or in this context, aging out of foster care. Transitions are internal psychological reactions to external changes. Substantial changes tend to yield more difficult transitions. Though the framework was originally developed for use in organizational management, it has been applied to other settings [25,26]. While it has not generally been applied to child welfare, one study examined its use with 34 Romanian foster youth leaving foster care to understand their social and psychological transitions and how they were both distinct and interconnected [27]. This study used the framework to help service providers, rather than the youth themselves, recognize the transition process for youth. To date, there has been little to no empirical research in which youth were taught about the framework to give them insight into their own experience of changes and potentially develop coping strategies as they enter and exit foster care.

The theory of change using this framework has several components to help youth cope with change. First, it provides youth with tools to recognize and understand their own internal transition responses and how the related behaviors may be received by others. This helps them to make sense of their own behaviors and anticipate their reactions, opening opportunities for changing their behavior. Second, it helps youth to normalize their emotional reactions to external change and increase their own patience as they adjust. For example, the framework includes a concept called “The Marathon Effect”, which explains that people will move at different paces toward a similar end or goal, that coping with change takes time, and there will be moments in which it feels like they are making faster progress than at other times. Understanding that setting goals works best after they have allowed themselves to grieve the past sets them up for success regarding those goals. Finally, the framework involves reinforcing the concepts and processes of the adults in their lives. This includes training their social workers/caseworks and foster parents and allows them to reinforce new learning and behaviors amongst the youth. Managing their own emotions and behaviors, in turn, can lead to more stable relationships at home and in the workplace.

### 1.6. The Present Study

This exploratory study examined how youth who were trained in the Transitions Framework fared 18 months later in three key wellbeing outcomes that historically are challenging for this group: social support, educational involvement, and employment. The findings presented here draw from the third and final wave of data collection from a multi-year evaluation of the Transitions Framework implemented in a non-profit U.S. Midwestern foster care provider (hereon referred to as “the agency”). The agency integrated the Bridges Transitions Framework into their programming to prepare adolescents in foster care adjust to their transitions into foster care, as well as to prepare older youth to age out of the system as young adults. Unlike other prior work, in addition to service providers, foster youth and their foster parents were trained.

### 1.7. Training Components and Application of Transitions Framework

Transitions Framework trainings aimed to help participants normalize emotional responses to change, become familiar their own internal transition process, and develop effective coping strategies. This knowledge is also important for the adults caring for youth, to understand what drives observable behaviors following a change in youth’s lives. The framework organizes reactions to change into phases called Endings, the Neutral Zone, and New Beginnings [16]. Though this generally represents a particular order, an individual may encounter aspects of more than one phase simultaneously. Critical to the framework is the focus on process rather than goals and moving at one’s own natural pace through the phases. Participants learned to understand and recognize the three transitions phases in their own lives, as described below.

Endings are triggered by loss, which for foster youth often includes separation from family and friends, connection to community and school, a shift to a new living situation with different people and rules, or discharge from foster care. The internal response may be experienced as a range of emotions such as anger, mourning, regret, sadness, regret, or nostalgia and wistfulness. The framework encourages reflection on the past and cautions against asking youth to set goals or develop new plans during this time. Problems with adjustment can occur if the past is not honored, or if youth are urged to focus on future plans while they still grieve. In this phase, social workers and caregivers are encouraged to help youth sustain meaningful memories by engaging in discussions about family or past events and listening when youth broach the subject, without attempting to move the conversation to the present or future.

During the Neutral Zone, endings are honored, and while youth accept the reality of their new situation, they may still feel unsettled in their living arrangement, ambivalent about new commitments, and untrusting of new relationships. In this phase, youth may exhibit disruptive or resistant behaviors or strong emotional reactions to people and situations. Caregivers and social workers are encouraged to exercise patience and recognize the underlying feelings that drive those actions rather than responding to the behaviors.

Finally, in New Beginnings, youth can imagine the future, establish goals, and embark on new relationships. This phase is marked by confidence and renewal. This is the phase where most people want to be, but, according to Bridges (2009) [16], they cannot attain it without first experiencing the first two phases. Here, foster parents and social workers help youth identify new paths toward goals and celebrate successes.

### 1.8. Implementation

The agency worked with a Transitions Framework expert consultant who conducted several two-day interactive trainings with all 10 agency social workers and supervisors, using a “train-the-trainer” approach. The social workers then conducted trainings with foster parents and adolescent foster youth served through the agency. The trainings were comprised of small groups (5–8 youths and their foster parents) and were conducted in four-session sequences, repeatedly offered over the course of the study. A rolling enrollment allowed youth aged as young as 13 (though the youngest participant was 15 years old) to participate shortly after they were admitted to foster care over the course of three years. Some of the trained youth participated in future trainings as “Transitions Coaches”, co-leading new trainings and sharing their own transitions experiences to demonstrate how the framework worked in real situations.

The structure was highly interactive. Participants mapped their transition processes on paper, shared stories, and practiced assessing their own and others’ transitional phases. Both youth and foster parents were provided with concrete behaviors and strategies to experiment with at home and then discuss in subsequent sessions for feedback and direction. For their part, foster parents were encouraged to value and honor the losses youth endured and to identify triggers and recognize underlying factors that drive challenging youth behaviors. They then practiced effective response strategies taught in the trainings.

To support continued use of the framework, the social workers integrated the transitions concepts in meetings with foster parent meetings and during youth programming. When behavioral problems were discussed, social workers asked youth and foster parents to first identify recent and past changes that might contribute to the behaviors and to identify the youth’s stage of transitions.

## 2. Methods

The findings here report on findings 18 months after foster youth were initially trained in the Transitions Framework. In this exploratory study, participants were interviewed in person, answering questions about their current living situation, future plans, social support, education, and employment.

### 2.1. Human Subjects Review

The research activities for this study were reviewed and approved by two research ethics boards: the primary investigator’s university IRB as well as the county human services IRB that oversaw the children in the foster care agency. All participants underwent an informed consent process; for minors, legal guardian consent and age-appropriate child assents were obtained prior to enrolling in the study.

### 2.2. Sampling

The participants were drawn from a pool of 54 youths who were trained in the Transitions Framework and took a post-test 6–9 months later. The original training included 63 youths who provided pre-test baseline data. Eighteen months post-training, all 54 youths were invited to participate in a final interview, and 35 (64.8%) enrolled. Most of the 19 youths who did not participate in the final interview (84%, *n* = 16) did not respond to invitations. For the remaining three, their legal guardians declined continued involvement for the youth.

While there are no data available across all categories for those who did not participate in the final interview, there were some demographic and placement data available that did not reveal substantial differences. An examination of demographics and placement information revealed that those who did not continue to third wave of data collection (Wave 3) were quite similar to those who participated, with very slight variations (6% or less) in race distributions, gender breakdown, age, proportion who were discharged, age at discharge, and reason for placement. For the 19 who did not continue, due to very small numbers in each subcategory and for confidentiality reasons, the specific subcategory breakdowns are not provided here.

### 2.3. Measurement and Data Collection

The data were collected to assess indicators of stability 18 months later. This study used the Medical Outcomes Study Social Support Survey (MOS) and agency administrative data from the youth’s placement records for demographic and placement information.

#### 2.3.1. Medical Outcomes Study Social Support Survey (MOS)

The MOS is a 19-item standardized tool found to be reliable and valid and has been used with foster youth in prior research [28]. It contains four social support subscales, including emotional-informational, tangible, affectionate, and positive social interaction. These were assessed with a five-point Likert scale, in which a score of 5 represents the most support (“all of the time”) and 1 represents the least support (“none of the time”).

#### 2.3.2. Administrative Data

Placement data were extracted from the youth’s placement records collected when the youth first enrolled in the study. These were data that were input into the data system contemporaneously by social service providers. To protect privacy, a data request for the following variables was submitted, and the foster agency staff pulled the information for the research: placement information included prior placement history, placement reasons, and permanency plans. For those who left foster care, discharge data that were routinely tracked and stored in the foster care database system were entered at the time of discharge, and information was pulled by agency staff. For those remaining in care 18 months later, placement records were used to collect updated information. These data included discharge date (if relevant), employment and educational status, living arrangement plans, and current living situation.

#### 2.3.3. Open-Ended Question

As part of the interview, participants were asked to share whether or not the transitions training continued to make a difference to them (and if so, in what ways) and to provide an example.

### 2.4. Analysis

Descriptive statistics were used to analyze the primary data from the 34 participants on employment status, living situation, educational status, the MOS social support responses, and demographics. The open-ended question was not intended to collect extensive or in-depth qualitative data but rather shed some light on whether or not the participants were still thinking about or using transitions concepts and if they perceived it as useful. The responses were fairly brief and organized according to the type of example they provided (if any) using a content analysis approach that assessed specific words and phrases in line with the type of impact.

## 3. Findings

### 3.1. Participants

All 35 youths were admitted to their most current placement as adolescents—at 16 years old on average. By 18 months post-training, 57% (*n* = 20) were discharged. The youth averaged 17 years old at the start of the study and 18.5 years old at Wave 3. Gender identity was nearly split between male and female, with one youth who identified as transgender. About three-fourths (74.3%) of the youth had a prior placement, and most (92.5%) were in non-relative foster care at the start of the study (Table 1). Consistent with national figures, the most common reason for placement was neglect (68.6%), followed by physical abuse (54.3%) and parental substance abuse (48.6%). Seventy percent of the youth had a permanency plan to remain in care until they aged out at 18 or 21, though a notable minority had plans for family reunification (15.8%). A large share of the youth had some visits with parents, with 55% occurring often. However, nearly a third did not have any parental visits

On average, the participants were 18.5 years old, and those discharged were 18.6 years old (Table 2). In terms of the racial and ethnic breakdown, it is not surprising that the foster care group is more ethnically diverse than national rates. This reflects the racial disproportionalities that exist in foster care nationally and even more heavily in the midwestern state where this study was conducted, where Black children are twice as likely as White children to enter a child welfare placement [29].

### 3.2. Still in Care, Plans for Discharge

Fifteen of the youth were still in care. Seven were scheduled to exit care within the year. Another six expected to leave within the next two years, and two planned to remain in care until they were 21. About three-quarters (*n* = 11) of the youth planned to enroll in college or technical school upon discharge, while two hoped to enroll in the military and two wanted to finish high school. In terms of employment plans, which could coincide with educational plans, eight youths planned to continue working at their current job and six expected to look for a job; the remaining youth was unsure.

### 3.3. Post-Discharge

Post-discharge, we examined the status of the participants’ education, employment, and relationship status (Table 3). They had a high rate of educational enrollment (80%) compared to 43% of 19-year-olds in the same state [30]. At this time, 5% were married and 10% were cohabitating.

Looking at high school or GED, 74.3% were enrolled, reflecting some of the younger participants who were still living in care. Post-discharge, only 35% of the participants reported employment, likely a factor of school enrollment.

Lastly, we turn to the measures of perceived social support. The MOS survey provided four scales to assess tangible, emotional, social, and affectionate support (Table 4). One the whole, the youth reported ratings in the mid to higher end of the five-point range. The mean scale scores ranged from 3.7 (tangible support) to 4.1 (positive social interaction and affection).

While the data cannot tell us whether transitions explicitly played a part in these high social support scores, the open-ended question responses aligned with the MOS results. All the participants named at least one aspect of the Transitions Framework training that they believed they benefitted from and retained 18 months later. The most common category and one in which most of the participates referenced regarded managing their emotions and the resulting impact on relationships. One participant explained the importance of understanding the root causes of their feelings.

“With Transitions, it has helped me understand like how I’m feeling and what I can do because I’m feeling that certain way and I don’t always have to blow up because I’m mad. I might be mad, but there’s something deeper that’s making me mad”.

Others used their new self-knowledge to temper how they kept lines of communication open with others when they were unhappy.

“It really helped me a lot knowing when I was angry, frustrated, and grieving. I know how to communicate that towards to people, instead of usually what I do is shut down and be off on my own”.

Several participants specifically named learning about the “Marathon Effect” as useful to them. Understanding the marathon effect made some youth feel better equipped to understand where others were and how to speak with them about their transitions,

“The marathon effect…I loved the idea that you don’t have to be rushed into anything, like you don’t have to feel like you should be at a certain point”.

Another explained, “The marathon effect is like if someone’s in Endings, you don’t necessarily be like, Oh, everything’s going to be okay, everything’s going to be good. Because they’re not ready to look at that kind of stuff yet”.

One component of the Transitions training is helping youth recognize the difference between reacting versus responding to stressful situations. Reacting, they learned, is behaving in a heated way without thought of consequence, whereas responding is a more thoughtful and careful way of responding. One participant shared,

“And then (the Transitions program) taught me like how to actually control my emotions, too. Well, Transitions mostly helped me on my anger, because before I was in the Transitions (program), I had a lot of anger issues. Like, if I get mad at someone, I pretty much get into a fight. I just learned that, you know, by fighting him, like by fighting a person like this is bad, it’s only going to make the problem bigger”.

## 4. Limitations

This study has several limitations. As an exploratory study, it does not include a control group, which would allow for the investigation of the counterfactual: what outcomes might look like in the absence of exposure to the intervention. To truly claim scientific evidence of change, there must be some sort of control group that offers a reasonable opportunity to compare based on key variables and is a way to rule out extraneous competing variables. Without a control group, it cannot be claimed that the framework was responsible for the findings. The sample size is small and represents youth from a single agency, limiting both the degree of analysis that can be conducted and generalizability. It is possible that the more motivated youth or foster parents attended the trainings, leading to more positive findings, though all foster parents are required to maintain continuing education credits, which were an incentive beyond simple motivation.

## 5. Discussion

The Transitions Framework offers a new approach to addressing a key source of stress and anxiety for youth in non-relative foster care: critical and monumental life changes that are not in their control but can alter everything that is familiar to them. There is some evidence that the framework may have positive short-term impacts on youth perceptions of change [26]. This study examined whether there were differences in longer-term well-being outcomes for education, employment, housing, and social support. This study found that 80% of the young people who were discharged were enrolled in school. Such a small sample, of course, does not permit much extrapolation. However, this is a positive sign, especially when state-level data show that only 43% of similar-aged youth exiting care were enrolled in school [29,31].

All foster youth experience transitions that can be difficult if not traumatizing—for example, the beginning of the day they are first placed in out-of-home care, which yields long-lasting negative impacts on their early adulthood [8,18]. Implemented in a foster care context, the Transitions Framework aims to help youth and their caregivers understand the link between external change, the internal experience of it (transitions) and how this influences behavior, emotions, and relationships [16]. The framework offers clear concepts in lay language that are accessible to young people, as well as concrete strategies to apply them.

Employment post-discharge was 35%, while it was 46% in the rest of the state among care leavers. This may be an artifact of their high level of school enrollment (80% overall; 50% post-secondary), though it cannot be determined with these data.

The youth, including those discharged, reported fairly positive support across all four MOS scales. The responses to the open-ended question indicate that participants were applying learnings from the framework in their everyday lives, contributing to better social relationships. We know that social support and connectedness have been a longstanding concern amongst foster care leavers who aged out [8,22,32]. While this study was not able to determine causal effect, as the first foster care agency to apply this framework throughout their programming, the findings are at least promising and point to the value of further research in a larger, controlled study.

## 6. Conclusions

It is evident from the extant research in the U.S. that the child welfare system needs to improve the stability and quality of care for youth residing in out-of-home placements and better prepare them for their transition to adulthood (e.g., [9]). This is a longstanding intractable problem in child welfare, yet there exist scant evidenced-based interventions that have addressed the issue. In particular, it is critical to attend to foster youth’s educational, employment, and socio-emotional needs as they move toward discharge and independent living. At the same time, youth need strategies to manage the emotional trauma of admission into care and repeated placement moves during placement. In turn, this may help them build healthier and sustained relationships. Educating youth and those who care for them about the process of adjusting to transitions can normalize youth reactions to change, raise personal awareness about past traumas and emotional responses, foster psychological wellbeing, and enhance healthy relationships [11,16]. Introducing these strategies prior to discharge, in conjunction with programming traditional independent living skills, allows youth to practice new ways of coping while they are still in a safe setting.

## Figures and Tables

**Table 1:** Placement variables.

Variable	Percent
**Non-relative placement** (at time of programming)	92.5
**Reason for placement** *	
Neglect	68.6
Physical abuse	54.3
Parental substance abuse	48.6
Other reason	20.0
**Permanency plan**	
Reunification	15.8
Remain in care until age out	71.4
Adoption	5.7
Other	7.1
**Parental visits**	
Frequent	54.2
Occasionally/irregular	14.4
None	31.4
**Prior placement**	74.3
Age at prior placement (years)	10.8

* Does not sum to 100% because they can fall into more than one category.

**Table 2:** Ethnicity and age *.

Demographic	%
African Amer./Black/African	55.4
Asian or Pacific Islander	8.6
Caucasian	20.0
Native American	0.0
Hispanic	10.8
Multiracial *	10.8
Mean age (years)	18.5
Age range (years)	16.4–19.8

* Columns do not add to 100% due to overlapping categories.

**Table 3:** Education and employment.

	All	Discharged
	*n* = 35	*n* = 20
Education: Enrolled in school	88.6%	80%
Enrolled in high school or GED program	74.3%	30%
Enrolled in post-secondary ed./vocation	14.3%	50%
Employment (part-time, post-discharge)	-	35%

**Table 4:** Perceived social support (*n* = 35).

Subscales and Items How Often Do You Have Someone to…	Mean Score *
Emotional/Informational Support, Scale Score	3.8
listen to you	3.9
confide in	4.0
share your worries with	3.5
understand your problems	3.7
give you information	3.7
give you advice you really want	4.1
turn to for suggestions	4.0
Tangible Support, Scale Score	3.7
help you if you were confined to bed	3.4
take you to the doctor	3.8
prepare your meals if you were unable to do it yourself	3.9
help with daily chores if you were sick	3.7
Positive Social Interaction Support, Scale Score	4.1
have a good time with	4.2
get together with for relaxation	3.8
do something enjoyable with	4.2
Affectionate Support, Scale Score	4.1
shows you love and affection	4.1
love you and make you feel wanted	4.1
who hugs you	4.2

* 1 = none of the time; 5 = all the time.

## Data Availability

The dataset presented in this article are not readily available because of privacy and ethic restrictions in regard to the study participants. Requests to access the datasets should be directed to nesm3326@stthomas.edu.
